# *N*-(4-Methyl­phen­yl)-*N*′-phenyl­butane­diamide monohydrate

**DOI:** 10.1107/S1600536811018915

**Published:** 2011-05-25

**Authors:** B. S. Saraswathi, Sabine Foro, B. Thimme Gowda

**Affiliations:** a Department of Chemistry, Mangalore University, Mangalagangotri 574 199, Mangalore, India; b Institute of Materials Science, Darmstadt University of Technology, Petersenstrasse 23, D-64287 Darmstadt, Germany

## Abstract

In the title hydrate, C_17_H_18_N_2_O_2_·H_2_O, the dihedral angles formed by the aromatic rings of the benzene and methyl­benzene groups with the mean planes of the attached NH—C(O)—CH_2_ fragments are 12.6 (4) and 23.3 (3)°, respectively, while that between the two aromatic rings is 73.7 (2)°. In the crystal, the water mol­ecule accepts two and makes two hydrogen bonds. The mol­ecules are packed into layers parallel to (101) by O—H⋯O and N—H⋯O hydrogen-bonding inter­actions.

## Related literature

For our study of the effect of substituents on the structures of *N*-(ar­yl)-amides, see: Gowda *et al.* (2000 ▶); Saraswathi *et al.* (2011**a* ▶,b* ▶) and on the structures of *N*-(ar­yl)-methane­sulfonamides, see: Gowda *et al.* (2007 ▶). For restrained geometry, see: Nardelli (1999 ▶).
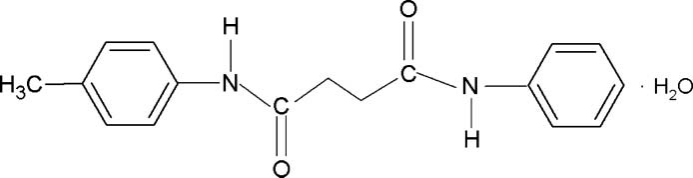


## Experimental

### 

#### Crystal data

C_17_H_18_N_2_O_2_·H_2_O
*M* *_r_* = 300.35
Monoclinic, 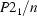

*a* = 15.242 (4) Å
*b* = 4.905 (1) Å
*c* = 21.540 (5) Å
β = 102.90 (2)°
*V* = 1569.7 (6) Å^3^
*Z* = 4
Mo *K*α radiation
μ = 0.09 mm^−1^
*T* = 293 K
0.44 × 0.12 × 0.08 mm

#### Data collection

Oxford Diffraction Xcalibur diffractometer with a Sapphire CCD detector
Absorption correction: multi-scan (*CrysAlis RED*; Oxford Diffraction, 2009 ▶) *T*_min_ = 0.962, *T*_max_ = 0.993
5068 measured reflections
2805 independent reflections
1356 reflections with *I* > 2σ(*I*)
*R*_int_ = 0.058

#### Refinement

*R*[*F* ^2^ > 2σ(*F* ^2^)] = 0.117
*wR*(*F* ^2^) = 0.239
*S* = 1.16
2805 reflections
211 parameters
5 restraints
H atoms treated by a mixture of independent and constrained refinement
Δρ_max_ = 0.35 e Å^−3^
Δρ_min_ = −0.31 e Å^−3^

### 

Data collection: *CrysAlis CCD* (Oxford Diffraction, 2009 ▶); cell refinement: *CrysAlis RED* (Oxford Diffraction, 2009 ▶); data reduction: *CrysAlis RED*; program(s) used to solve structure: *SHELXS97* (Sheldrick, 2008 ▶); program(s) used to refine structure: *SHELXL97* (Sheldrick, 2008 ▶); molecular graphics: *PLATON* (Spek, 2009 ▶); software used to prepare material for publication: *SHELXL97*.

## Supplementary Material

Crystal structure: contains datablocks I, global. DOI: 10.1107/S1600536811018915/tk2744sup1.cif

Structure factors: contains datablocks I. DOI: 10.1107/S1600536811018915/tk2744Isup2.hkl

Supplementary material file. DOI: 10.1107/S1600536811018915/tk2744Isup3.cml

Additional supplementary materials:  crystallographic information; 3D view; checkCIF report

## Figures and Tables

**Table 1:** Hydrogen-bond geometry (Å, °)

*D*—H⋯*A*	*D*—H	H⋯*A*	*D*⋯*A*	*D*—H⋯*A*
N1—H1*N*⋯O2^i^	0.85 (2)	2.06 (2)	2.895 (6)	168 (6)
N2—H2*N*⋯O3^ii^	0.86 (2)	2.15 (2)	2.992 (6)	169 (5)
O3—H31⋯O1	0.85 (2)	1.93 (2)	2.762 (6)	165 (5)
O3—H32⋯O3^iii^	0.85 (2)	2.03 (2)	2.858 (5)	166 (5)

Symmetry codes: (i) 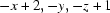; (ii) 
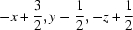
; (iii) 
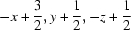
.

**Fig. 1.**
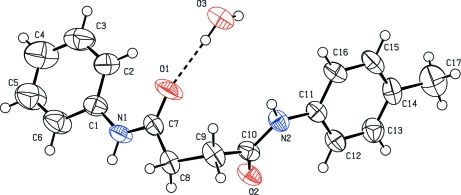
The asymmetric unit of the title compound showing atom labelling and with displacement ellipsoids drawn at the 50% probability level. The hydrogen bond is shown as a dashed line.

**Fig. 2.**
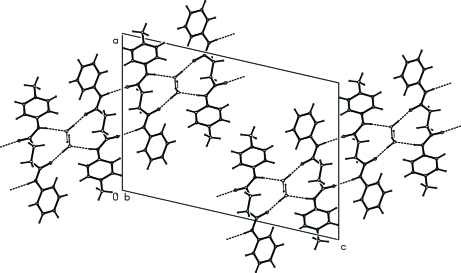
A partial packing diagram of the title compound viewed in projection down the b direction, showing the hydrogen bonding scheme with dashed lines.

## supplementary crystallographic information

### Comment

The amide and sulfonamide moieties are important constituents of many biologically significant compounds. As a part of a study of the substituent effects on their structures and other aspects of this class of compounds (Gowda *et al.*, 2000, 2007; Saraswathi *et al.*, 2011**a*,b*), in the present work, the structure of the title compound, isolated as a monohydrate has been determined (Fig. 1). The conformation of N—H and C═ O bonds in each C—NH—C(O)—C segment is *anti*, similar to that observed in *N*,*N*-bis(2-methylphenyl)- succinamide (II) (Saraswathi *et al.*, 2011*a*) and in *N*,*N*-bis(3-chlorophenyl)-succinamide (III) (Saraswathi *et al.*, 2011*b*).

The dihedral angle between the phenyl ring and the adjacent NH—C(O)—CH_2_ segment is 12.6 (4) ° and that between the 4-methylphenyl ring and the adjacent NH—C(O)—CH_2_ segment is 23.3 (3) °, compared to the values of 62.1 (2) ° formed between the benzene ring and the NH—C(O)—CH_2_ segment in the two halves of (II), and 32.8 (1) ° in (III). In the title compound, the dihedral angle between the two aromatic rings is 73.7 (2) °. The crystal packing is stabilized through N1—H1N···O2, N2—H2N···O3, O3—H31···O1 and O3—H32···O3 hydrogen bonding (Table 1) and results in layers as shown in Fig.2.

### Experimental

Succinic anhydride (0.01 mol) in toluene (25 ml) was treated drop wise with aniline (0.01 mol) also in toluene (20 ml) with constant stirring. The resulting mixture was stirred for one hour and set aside for an additional hour at room temperature for completion of the reaction. The mixture was then treated with dilute hydrochloric acid to remove unreacted aniline. The resultant *N*-(phenyl)succinamic acid was filtered under suction and washed thoroughly with water to remove the unreacted succinic anhydride and succinic acid. The compound was recrystallized to constant melting point from ethanol.

The *N*-(phenyl)succinamic acid obtained was then treated with phosphorous oxychloride and excess of *p*-toluidine at room temperature with constant stirring. The resultant mixture was stirred for 4 h, kept aside for additional 6 h for completion of the reaction and poured slowly into crushed ice with constant stirring. It was kept aside for a day. The resultant solid, *N*-(phenyl),*N*-(4-methylphenyl)- succinamide monohydrate, was filtered under suction, washed thoroughly with water, dilute sodium hydroxide solution and finally with water. It was recrystallized to constant melting point from a mixture of acetone and chloroform.

Colorless needles were grown in a mixture of acetone and chloroform at room temperature.

### Refinement

The H atoms of the NH groups were located in a difference map and later restrained to the distance N—H = 0.86 (2) Å. The H atoms of the water molecule were located in difference map and were refined with the O—H and H—H distances restrained to 0.85 (2) Å and 1.365 Å, respectively, thus leading to the angle of 107 ° (Nardelli, 1999). The other H atoms were positioned in their idealized geometries using a riding model with aromatic C—H = 0.93 Å, methyl C—H = 0.96 Å and methylene C—H = 0.97 Å. All H atoms were refined with isotropic displacement parameters (set to 1.2 times of the *U*_eq_ of the parent atom).

### Crystal data

**Table:**

C_17_H_18_N_2_O_2_·H_2_O	*F*(000) = 640
*M**_r_* = 300.35	*D*_x_ = 1.271 Mg m^−^^3^
Monoclinic, *P*2_1_/*n*	Mo *K*α radiation, λ = 0.71073 Å
Hall symbol: -P 2yn	Cell parameters from 897 reflections
*a* = 15.242 (4) Å	θ = 2.6–27.8°
*b* = 4.905 (1) Å	µ = 0.09 mm^−^^1^
*c* = 21.540 (5) Å	*T* = 293 K
β = 102.90 (2)°	Needle, colourless
*V* = 1569.7 (6) Å^3^	0.44 × 0.12 × 0.08 mm
*Z* = 4	

### Data collection

**Table:**

Oxford Diffraction Xcalibur diffractometer with a Sapphire CCD detector	2805 independent reflections
Radiation source: fine-focus sealed tube	1356 reflections with *I* > 2σ(*I*)
graphite	*R*_int_ = 0.058
Rotation method data acquisition using ω scans	θ_max_ = 25.4°, θ_min_ = 2.9°
Absorption correction: multi-scan (*CrysAlis RED*; Oxford Diffraction, 2009)	*h* = −18→15
*T*_min_ = 0.962, *T*_max_ = 0.993	*k* = −5→4
5068 measured reflections	*l* = −25→21

### Refinement

**Table:**

Refinement on *F*^2^	Primary atom site location: structure-invariant direct methods
Least-squares matrix: full	Secondary atom site location: difference Fourier map
*R*[*F*^2^ > 2σ(*F*^2^)] = 0.117	Hydrogen site location: inferred from neighbouring sites
*wR*(*F*^2^) = 0.239	H atoms treated by a mixture of independent and constrained refinement
*S* = 1.16	*w* = 1/[σ^2^(*F*_o_^2^) + (0.0521*P*)^2^ + 3.417*P*] where *P* = (*F*_o_^2^ + 2*F*_c_^2^)/3
2805 reflections	(Δ/σ)_max_ < 0.001
211 parameters	Δρ_max_ = 0.35 e Å^−^^3^
5 restraints	Δρ_min_ = −0.31 e Å^−^^3^

### Special details

**Table:**

Experimental. CrysAlis RED (Oxford Diffraction, 2009) Empirical absorption correction using spherical harmonics, implemented in SCALE3 ABSPACK scaling algorithm.
Geometry. All e.s.d.'s (except the e.s.d. in the dihedral angle between two l.s. planes) are estimated using the full covariance matrix. The cell e.s.d.'s are taken into account individually in the estimation of e.s.d.'s in distances, angles and torsion angles; correlations between e.s.d.'s in cell parameters are only used when they are defined by crystal symmetry. An approximate (isotropic) treatment of cell e.s.d.'s is used for estimating e.s.d.'s involving l.s. planes.
Refinement. Refinement of *F*^2^ against ALL reflections. The weighted *R*-factor *wR* and goodness of fit *S* are based on *F*^2^, conventional *R*-factors *R* are based on *F*, with *F* set to zero for negative *F*^2^. The threshold expression of *F*^2^ > σ(*F*^2^) is used only for calculating *R*-factors(gt) *etc*. and is not relevant to the choice of reflections for refinement. *R*-factors based on *F*^2^ are statistically about twice as large as those based on *F*, and *R*- factors based on ALL data will be even larger.

### Fractional atomic coordinates and isotropic or equivalent isotropic displacement parameters (Å^2^)

**Table:**

	*x*	*y*	*z*	*U*_iso_*/*U*_eq_	
C1	1.1172 (4)	0.2471 (13)	0.3698 (3)	0.0477 (16)	
C2	1.0806 (5)	0.4142 (15)	0.3191 (3)	0.063 (2)	
H2	1.0187	0.4183	0.3031	0.076*	
C3	1.1363 (6)	0.5749 (17)	0.2923 (4)	0.080 (2)	
H3	1.1113	0.6843	0.2576	0.096*	
C4	1.2289 (6)	0.5780 (17)	0.3157 (4)	0.090 (3)	
H4	1.2656	0.6879	0.2971	0.108*	
C5	1.2648 (5)	0.4153 (18)	0.3667 (4)	0.089 (3)	
H5	1.3267	0.4150	0.3831	0.107*	
C6	1.2101 (4)	0.2525 (16)	0.3940 (3)	0.071 (2)	
H6	1.2354	0.1448	0.4289	0.085*	
C7	0.9812 (4)	−0.0135 (12)	0.3804 (3)	0.0426 (16)	
C8	0.9514 (3)	−0.2274 (13)	0.4215 (3)	0.0440 (16)	
H8A	0.9736	−0.1789	0.4659	0.053*	
H8B	0.9781	−0.4008	0.4144	0.053*	
C9	0.8499 (4)	−0.2600 (12)	0.4085 (3)	0.0487 (17)	
H9A	0.8356	−0.4171	0.4317	0.058*	
H9B	0.8272	−0.2944	0.3634	0.058*	
C10	0.8029 (4)	−0.0102 (12)	0.4276 (3)	0.0418 (16)	
C11	0.6627 (4)	0.2679 (12)	0.3937 (3)	0.0399 (15)	
C12	0.6588 (4)	0.3788 (13)	0.4515 (3)	0.0477 (17)	
H7	0.6988	0.3221	0.4884	0.057*	
C13	0.5948 (4)	0.5757 (14)	0.4546 (3)	0.0555 (18)	
H13	0.5927	0.6499	0.4940	0.067*	
C14	0.5343 (4)	0.6647 (12)	0.4013 (4)	0.0497 (17)	
C15	0.5394 (4)	0.5532 (14)	0.3437 (3)	0.0569 (19)	
H15	0.4995	0.6109	0.3068	0.068*	
C16	0.6028 (4)	0.3567 (13)	0.3398 (3)	0.0513 (17)	
H16	0.6051	0.2835	0.3004	0.062*	
C17	0.4654 (5)	0.8807 (15)	0.4067 (4)	0.086 (3)	
H17A	0.4060	0.8100	0.3904	0.103*	
H17B	0.4746	1.0382	0.3824	0.103*	
H17C	0.4719	0.9309	0.4505	0.103*	
N1	1.0664 (3)	0.0735 (11)	0.4004 (2)	0.0475 (14)	
H1N	1.095 (3)	−0.005 (11)	0.4340 (17)	0.057*	
N2	0.7246 (3)	0.0584 (10)	0.3870 (2)	0.0421 (13)	
H2N	0.716 (4)	−0.031 (10)	0.3519 (16)	0.051*	
O1	0.9311 (3)	0.0725 (11)	0.3319 (2)	0.0762 (16)	
O2	0.8342 (3)	0.1165 (8)	0.47672 (18)	0.0509 (12)	
O3	0.7946 (3)	0.3120 (9)	0.2425 (2)	0.0596 (13)	
H31	0.839 (3)	0.268 (11)	0.273 (2)	0.071*	
H32	0.773 (4)	0.460 (8)	0.253 (3)	0.071*	

### Atomic displacement parameters (Å^2^)

**Table:**

	*U*^11^	*U*^22^	*U*^33^	*U*^12^	*U*^13^	*U*^23^
C1	0.054 (4)	0.044 (4)	0.040 (4)	−0.001 (4)	0.001 (3)	−0.004 (3)
C2	0.069 (5)	0.070 (5)	0.049 (4)	0.004 (4)	0.011 (4)	0.007 (4)
C3	0.102 (7)	0.073 (6)	0.063 (5)	0.011 (6)	0.011 (5)	0.020 (5)
C4	0.100 (7)	0.078 (6)	0.084 (6)	−0.026 (6)	0.003 (5)	0.008 (5)
C5	0.071 (5)	0.099 (7)	0.088 (6)	−0.024 (5)	−0.004 (5)	0.025 (6)
C6	0.057 (5)	0.076 (6)	0.068 (5)	−0.015 (4)	−0.009 (4)	0.015 (4)
C7	0.039 (3)	0.043 (4)	0.043 (4)	0.008 (3)	0.002 (3)	−0.005 (3)
C8	0.036 (3)	0.039 (4)	0.053 (4)	0.003 (3)	0.003 (3)	−0.008 (3)
C9	0.042 (4)	0.032 (4)	0.065 (4)	−0.002 (3)	−0.004 (3)	−0.011 (3)
C10	0.036 (3)	0.040 (4)	0.044 (4)	−0.009 (3)	−0.003 (3)	0.005 (3)
C11	0.037 (3)	0.034 (4)	0.045 (4)	−0.004 (3)	0.001 (3)	−0.003 (3)
C12	0.037 (3)	0.052 (4)	0.049 (4)	0.002 (3)	−0.001 (3)	−0.001 (4)
C13	0.047 (4)	0.060 (5)	0.058 (4)	−0.001 (4)	0.009 (3)	−0.007 (4)
C14	0.036 (4)	0.031 (4)	0.083 (5)	−0.002 (3)	0.015 (4)	0.003 (4)
C15	0.041 (4)	0.054 (5)	0.067 (5)	0.009 (4)	−0.004 (3)	0.013 (4)
C16	0.046 (4)	0.054 (4)	0.047 (4)	0.003 (4)	−0.004 (3)	0.005 (3)
C17	0.072 (5)	0.071 (6)	0.113 (7)	−0.001 (5)	0.016 (5)	0.004 (5)
N1	0.043 (3)	0.052 (4)	0.040 (3)	0.002 (3)	−0.005 (2)	0.012 (3)
N2	0.036 (3)	0.043 (3)	0.041 (3)	−0.002 (3)	−0.004 (2)	−0.009 (3)
O1	0.051 (3)	0.100 (4)	0.062 (3)	0.001 (3)	−0.020 (2)	0.032 (3)
O2	0.050 (3)	0.047 (3)	0.046 (2)	0.006 (2)	−0.010 (2)	−0.005 (2)
O3	0.060 (3)	0.053 (3)	0.056 (3)	0.009 (2)	−0.008 (2)	0.005 (2)

### Geometric parameters (Å, °)

**Table:**

C1—C2	1.380 (8)	C10—O2	1.227 (6)
C1—C6	1.396 (8)	C10—N2	1.356 (6)
C1—N1	1.410 (8)	C11—C12	1.372 (8)
C2—C3	1.377 (10)	C11—C16	1.378 (7)
C2—H2	0.9300	C11—N2	1.424 (7)
C3—C4	1.388 (10)	C12—C13	1.385 (8)
C3—H3	0.9300	C12—H7	0.9300
C4—C5	1.370 (10)	C13—C14	1.373 (8)
C4—H4	0.9300	C13—H13	0.9300
C5—C6	1.376 (9)	C14—C15	1.374 (9)
C5—H5	0.9300	C14—C17	1.515 (9)
C6—H6	0.9300	C15—C16	1.381 (8)
C7—O1	1.222 (6)	C15—H15	0.9300
C7—N1	1.343 (7)	C16—H16	0.9300
C7—C8	1.507 (8)	C17—H17A	0.9600
C8—C9	1.517 (7)	C17—H17B	0.9600
C8—H8A	0.9700	C17—H17C	0.9600
C8—H8B	0.9700	N1—H1N	0.85 (2)
C9—C10	1.522 (8)	N2—H2N	0.859 (19)
C9—H9A	0.9700	O3—H31	0.854 (19)
C9—H9B	0.9700	O3—H32	0.847 (19)
			
C2—C1—C6	118.8 (7)	O2—C10—C9	121.7 (5)
C2—C1—N1	124.2 (6)	N2—C10—C9	115.1 (5)
C6—C1—N1	117.0 (6)	C12—C11—C16	119.0 (6)
C3—C2—C1	119.6 (7)	C12—C11—N2	122.9 (5)
C3—C2—H2	120.2	C16—C11—N2	118.1 (5)
C1—C2—H2	120.2	C11—C12—C13	119.6 (6)
C2—C3—C4	121.7 (7)	C11—C12—H7	120.2
C2—C3—H3	119.1	C13—C12—H7	120.2
C4—C3—H3	119.1	C14—C13—C12	122.0 (6)
C5—C4—C3	118.5 (8)	C14—C13—H13	119.0
C5—C4—H4	120.8	C12—C13—H13	119.0
C3—C4—H4	120.8	C13—C14—C15	117.8 (6)
C4—C5—C6	120.6 (8)	C13—C14—C17	120.4 (7)
C4—C5—H5	119.7	C15—C14—C17	121.8 (6)
C6—C5—H5	119.7	C14—C15—C16	120.9 (6)
C5—C6—C1	120.8 (7)	C14—C15—H15	119.5
C5—C6—H6	119.6	C16—C15—H15	119.5
C1—C6—H6	119.6	C11—C16—C15	120.7 (6)
O1—C7—N1	122.6 (6)	C11—C16—H16	119.7
O1—C7—C8	122.0 (5)	C15—C16—H16	119.7
N1—C7—C8	115.4 (5)	C14—C17—H17A	109.5
C7—C8—C9	113.1 (5)	C14—C17—H17B	109.5
C7—C8—H8A	109.0	H17A—C17—H17B	109.5
C9—C8—H8A	109.0	C14—C17—H17C	109.5
C7—C8—H8B	109.0	H17A—C17—H17C	109.5
C9—C8—H8B	109.0	H17B—C17—H17C	109.5
H8A—C8—H8B	107.8	C7—N1—C1	129.4 (5)
C8—C9—C10	112.8 (5)	C7—N1—H1N	114 (4)
C8—C9—H9A	109.0	C1—N1—H1N	116 (4)
C10—C9—H9A	109.0	C10—N2—C11	128.4 (5)
C8—C9—H9B	109.0	C10—N2—H2N	113 (4)
C10—C9—H9B	109.0	C11—N2—H2N	119 (4)
H9A—C9—H9B	107.8	H31—O3—H32	107 (3)
O2—C10—N2	123.2 (6)		
			
C6—C1—C2—C3	2.1 (10)	C12—C13—C14—C15	−0.5 (10)
N1—C1—C2—C3	−179.6 (6)	C12—C13—C14—C17	−179.9 (6)
C1—C2—C3—C4	−1.3 (12)	C13—C14—C15—C16	0.5 (10)
C2—C3—C4—C5	0.1 (13)	C17—C14—C15—C16	179.9 (6)
C3—C4—C5—C6	0.1 (13)	C12—C11—C16—C15	−0.3 (9)
C4—C5—C6—C1	0.7 (13)	N2—C11—C16—C15	177.9 (5)
C2—C1—C6—C5	−1.9 (11)	C14—C15—C16—C11	−0.1 (10)
N1—C1—C6—C5	179.7 (7)	O1—C7—N1—C1	−7.0 (10)
O1—C7—C8—C9	−16.9 (8)	C8—C7—N1—C1	172.5 (6)
N1—C7—C8—C9	163.6 (5)	C2—C1—N1—C7	16.7 (10)
C7—C8—C9—C10	−67.0 (7)	C6—C1—N1—C7	−165.0 (6)
C8—C9—C10—O2	−40.1 (8)	O2—C10—N2—C11	−4.1 (9)
C8—C9—C10—N2	140.3 (5)	C9—C10—N2—C11	175.4 (5)
C16—C11—C12—C13	0.3 (9)	C12—C11—N2—C10	−21.1 (9)
N2—C11—C12—C13	−177.9 (5)	C16—C11—N2—C10	160.7 (6)
C11—C12—C13—C14	0.1 (9)		

### Hydrogen-bond geometry (Å, °)

**Table:**

*D*—H···*A*	*D*—H	H···*A*	*D*···*A*	*D*—H···*A*
N1—H1N···O2^i^	0.85 (2)	2.06 (2)	2.895 (6)	168 (6)
N2—H2N···O3^ii^	0.86 (2)	2.15 (2)	2.992 (6)	169 (5)
O3—H31···O1	0.85 (2)	1.93 (2)	2.762 (6)	165 (5)
O3—H32···O3^iii^	0.85 (2)	2.03 (2)	2.858 (5)	166 (5)

Symmetry codes: (i) −*x*+2, −*y*, −*z*+1; (ii) −*x*+3/2, *y*−1/2, −*z*+1/2; (iii) −*x*+3/2, *y*+1/2, −*z*+1/2.

## References

[bb1] Gowda, B. T., Foro, S. & Fuess, H. (2007). *Acta Cryst.* E**63**, o2570.

[bb2] Gowda, B. T., Svoboda, I. & Fuess, H. (2000). *Z. Naturforsch. Teil A*, **55**, 779–790.

[bb3] Nardelli, M. (1999). *J. Appl. Cryst.* **32**, 563–571.

[bb4] Oxford Diffraction (2009). *CrysAlis CCD* and *CrysAlis RED* Oxford Diffraction Ltd, Yarnton, England.

[bb5] Saraswathi, B. S., Foro, S. & Gowda, B. T. (2011*a*). *Acta Cryst.* E**67**, o607. 10.1107/S1600536811004442 PMC3052079 21522364

[bb6] Saraswathi, B. S., Foro, S. & Gowda, B. T. (2011*b*). *Acta Cryst.* E**67**, o966. 10.1107/S1600536811010440 PMC3099800 21754229

[bb7] Sheldrick, G. M. (2008). *Acta Cryst.* A**64**, 112–122. 10.1107/S0108767307043930 18156677

[bb8] Spek, A. L. (2009). *Acta Cryst.* D**65**, 148–155. 10.1107/S090744490804362X PMC2631630 19171970

